# Structure–Property Correlations in Aqueous Binary Na^+^/K^+^–CH_3_COO^–^ Highly Concentrated Electrolytes

**DOI:** 10.1021/acs.jpcc.3c01017

**Published:** 2023-05-13

**Authors:** Shahid Khalid, Nicolò Pianta, Simone Bonizzoni, Chiara Ferrara, Roberto Lorenzi, Alberto Paleari, Patrik Johansson, Piercarlo Mustarelli, Riccardo Ruffo

**Affiliations:** † Department of Materials Science, University of Milano-Bicocca, via Cozzi 55, 20125 Milano, Italy; ‡ Department of Physics, Chalmers University of Technology, SE-41296 Göteborg, Sweden; § National Reference Center for Electrochemical Energy Storage (GISEL), Consorzio Interuniversitario Nazionale per la Scienza e Tecnologia dei Materiali (INSTM), 50121 Firenze, Italy

## Abstract

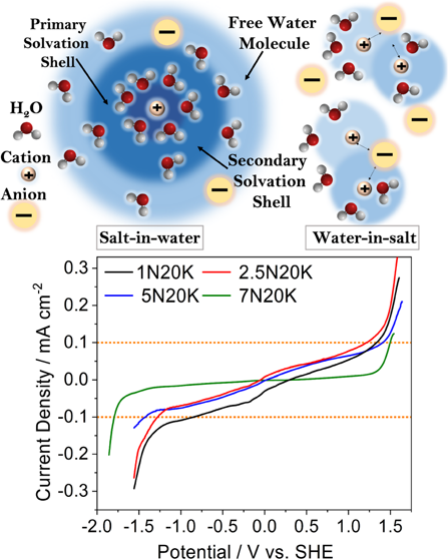

Highly concentrated aqueous binary solutions of acetate salts are promising systems for different electrochemical applications, for example, energy storage devices. The very high solubility of CH_3_COOK allows us to obtain water-in-salt electrolyte concentrations, thus reducing ion activity and extending the cathodic stability of an aqueous electrolyte. At the same time, the presence of Li^+^ or Na^+^ makes these solutions compatible with intercalation materials for the development of rechargeable alkaline-ion batteries. Although there is a growing interest in these systems, a fundamental understanding of their physicochemical properties is still lacking. Here, we report and discuss the physicochemical and electrochemical properties of a series of solutions based on 20 mol kg^–1^ CH_3_COOK with different concentrations of CH_3_COONa. The most concentrated solution, 20 mol kg^–1^ CH_3_COOK + 7 mol kg^–1^ CH_3_COONa, gives the best compromise between transport properties and electrochemical stability, displaying a conductivity of 21.2 mS cm^–1^ at 25 °C and a stability window of up to 3 V in “ideal” conditions, i.e., using a small surface area and highly electrocatalytic electrode in a flooded cell. Careful Raman spectroscopy analyses help to address the interaction network, the phase evolution with temperature, and the crystallization kinetics.

## Introduction

Water-in-salt electrolytes (WISEs) are a new class of ionic solutions, which belong to the wider category of solvent-in-salt (SIS) electrolytes,^1^ defined as concentrated solutions, in which the salt/solvent ratio is larger than unity in a weight or molar ratio.^2^ These systems have physicochemical properties (electrical conductivity, viscosity, electrochemical stability) that differ considerably from normal ionic solutions (molar salt/solvent ratio <0.2) and make them interesting for a number of electrochemical applications, in particular energy storage.^3^ Indeed, although the use of WISEs is recently raising interest in catalysis,^4^ electrochromic systems,^5^ and surface coatings,^6^ most of the work refers to applications in rechargeable batteries or supercapacitors.^7^ In this context, state-of-the-art devices use electrolytes based on liquid mixtures of carbonates, which contain flammable solvents, such as diethyl carbonate (DEC) or dimethyl carbonate (DMC).^8^ From the standpoint of cell safety, the best choice would be to use aqueous electrolytes, as water exhibits physicochemical properties that make it the ideal solvent in electrolyte mixtures. In fact, among polar solvents, water shows optimal trade-off between a high dielectric constant and low viscosity so that electrolytes can achieve higher conductivities than their organic counterparts. However, the use of water in high-specific-energy batteries is limited by its modest electrochemical stability window (ESW), especially on the cathodic side, which is affected by the presence of protons or positively polarized hydrogen atoms.

Water-in-salt electrolytes (WISEs) were developed with the aim to increase the ESW,^9^ allowing the development of high-energy aqueous devices.^10,11^ In WISEs, the increase of ESW is generally attributed to the reduced activity of water at such a high concentration, being all of the solvent molecules incorporated into the solvation shells,^12^ which results in large overpotentials for the water electrolysis.^13^ More recent studies, however, have shown that the correct determination of E-SW expansion requires special attention to experimental aspects^14^ and is influenced by the nature of the species formed at the interface, the study of which requires advanced characterization techniques.^15^

Many WISEs employ per-fluorinated sulfonyl imide salts, such as lithium (fluorosulfonyl)(trifluoromethanesulfonyl)imide (FTFSI),^16^ lithium (pentafluoroethane sulfonyl)(trifluoromethanesulfonyl)imide (PTFSI), lithium bis(trifluoromethanesulfonyl)imide (LiTFSI),^2^ sodium trifluoromethanesulfonate (NaOTF),^17^ sodium bis(fluorosulfonyl)imide (NaFSI),^18^ and potassium trifluoromethanesulfonate (KOTF).^19^ Despite the outstanding improvements in the corresponding ESWs, the environmental and economic concerns remain regarding the use of fluorinated salts. In particular, LiTFSI, which is widely used in LIBs, exhibits severe oral and dermal toxicities and can cause chronic aquatic toxicity.^12^ Furthermore, the limited availability and very high cost of these fluorinated salts practically nullify the advantages brought by superconcentration, making the practical implementation of WISEs in energy storage very challenging.^20,21^

Examples of cost-effective, highly soluble, and widely available salts are LiCl, CH_3_COONa (NaAc), and CH_3_COOK (KAc). In particular, KAc was recently investigated by our group^22^ and proposed in potassium-ion batteries (KIBs), and supercapacitors.^23^ An interesting aspect of KAc, which broadens the scope of its applications, is the possibility to couple it with Li, Na, and Zn salts to achieve binary electrolytes with salt concentrations above 20 mol kg^–1^,^24^ enabling the use of such systems in different battery chemistries. For instance, Chen et al. reported a mixed-cation electrolyte composed of 32 mol kg^–1^ KAc and 1 mol kg^–1^ (CH_3_COO)_2_Zn, allowing a (2.0 V) Zn-MnO_2_ aqueous rechargeable cell.^25^ Another interesting report features an LIB equipped with 32 mol kg^–1^ KAc with a 7 mol kg^–1^ CH_3_COOLi electrolyte, utilizing C-TiO_2_ and LiMnO_4_ as anode and cathode active materials, respectively.^12^ Even more interesting is the binary solution with sodium, given the low cost and wide distribution/availability of the raw materials. In this view, a sodium-ion battery was recently developed based on the use of a 32 mol kg^–1^ KAc with an 8 mol kg^–1^ NaAc solution coupled with NASICON-type electrodes.^26^ Despite their technological interest, the interactions between the components of these liquid phases, which underlie the increase in ESW, are little investigated both in the bulk and with respect to the electrochemical properties of the interfaces. Interactions among components are also crucial to understand thermal, rheological, and electrical properties, thus aiding the design of new electrolytes. Raman spectroscopy is a useful tool for both the determination of electrolyte structure and the study of ion/ion and ion/solvent interactions.^27^

Here, we investigated a binary system made of KAc and NaAc by keeping the KAc concentration constant at 20 mol kg^–1^ and increasing NaAc up to 7 mol kg^–1^. The physicochemical and electrochemical properties (thermal behavior, electrical conductivity, viscosity, and ESW) of the binary system, which are crucial to correctly address its application in aqueous sodium-ion batteries, were deeply investigated. In addition, the increased ESW and peculiar thermal properties were rationalized by studying the atomic interactions with Raman spectroscopy measurements performed both at different NaAc concentrations and in a wide temperature range.

## Experimental Section

### Electrolyte Preparation

Appropriate amounts of NaAc (99%, Alfa Aesar) and KAc (99%, Alfa Aesar) were dissolved in Milli-Q water (σ = 6.7 μS cm^–1^) under sonication. Keeping KAc constant at 20 mol kg^–1^ and varying the concentration of NaAc, four different electrolytes were prepared, namely, 1 mol kg^–1^ NaAc with 20 mol kg^–1^ KAc (1N20K), 2.5 mol kg^–1^ NaAc with 20 mol kg^–1^ KAc (2.5N20K), 5 mol kg^–1^ NaAc with 20 mol kg^–1^ KAc (5N20K), and 7 mol kg^–1^ NaAc with 20 mol kg^–1^ KAc (7N20K). All of the electrolytes were stored and used at room conditions. The solution density was measured by volumetric analysis, and the corresponding molar concentration was calculated. Table 1 reports the compositional parameters of the samples.

**Table 1:** Composition of NaAc/KAc/H_2_O Solutions

sample name	density (g cm^–3^)	molarity (mol dm^–3^)	mol NaAc (%)	mol KAc (%)	mol H_2_O (%)	wt NaAc (%)	wt KAc (%)	wt H_2_O (%)	n*^a^
1N20K	1.37	9.45	1.31	26.14	72.55	2.69	64.47	32.84	2.64
2.5N20K	1.39	9.87	3.20	25.64	71.16	6.48	61.96	31.56	2.46
5N20K	1.40	10.38	6.20	24.84	68.96	12.16	58.19	29.65	2.22
7N20K	1.41	10.76	8.48	24.24	67.28	16.23	55.50	28.27	2.06

a n* = ratio of the number of water molecules to the salt molecules.

### Viscosity Measurements

Viscosity measurements were performed with an MCR rheometer 102 (Anton Paar). A parallel plate (50 mm) setup was used to perform shear tests with shear rates in the range 10–300 s^–1^ with steady-state settings. The measurements were carried out at 10, 20, 30, and 40 °C.

### Thermal Studies

Differential scanning calorimetry (DSC) was performed using a DSC 1 Star (Mettler Toledo). STARe software was used for the evaluation of the data. The samples were analyzed in a temperature range from −120 to 90 °C. The following protocol was used: cooling from 30 to −120 °C at a rate of 1 °C min^–1^, followed by a 5 min isotherm at −120 °C, and finally by a heating step from −120 to 90 °C at a rate of 1 °C min^–1^. Initially, all of the samples were equilibrated at 30 °C for 30 min to ensure a completely liquid state. The heating/cooling rate of 1 °C min^–1^ was selected, following preliminary tests, as the best compromise to allow the system to undergo structural relaxation without excessively sacrificing sensitivity.

### Conductivity Measurements

Conductivity measurements were performed using electrochemical impedance spectroscopy (EIS) employing a dip probe cell with two platinum foils in a glass casing, with a cell constant of 1.04 cm^–1^. Impedance spectra were recorded in a frequency range from 1 Hz to 200 kHz with an amplitude of 25 mV. All of the samples were first degassed with nitrogen flux, and the measurement was done under N_2_ flux in the temperature range from 5 to 70 °C in a climatic chamber (Angelantoni, Italy). For all of the samples, the bulk ohmic resistance was recorded via the *x*-axis intercept at high frequencies in the Nyquist plots.

### Raman Spectroscopy Measurements

Raman spectra were collected in backscattering configuration using a Labram Dilor spectrometer (JobinYvon) and the 488 nm line of an Ar^+^ laser as an excitation source with a resolution of 2 cm^–1^ by three accumulations of 30 s of integration. The beam was focused on a circular spot through the optics of a microscope (Olympus), a long working distance objective with 50× magnification, and a numerical aperture of 0.60. Measurements in the temperature range from −150 to 20 °C were carried out by means of a cryostat working with liquid nitrogen flux and a programmable heater, with a final thermal stabilization within ±2 °C.

### Electrochemical Measurements

The ESW was estimated by linear sweep voltammetry (LSV) in a flooded three-electrode cell equipped with glassy carbon as working, platinum mesh as counter, and double junction SCE as reference electrodes (REs), respectively. A volume of 10 mL of electrolyte was used in each LSV measurement. The working electrode (WE) was a glassy carbon pin (3 mm diameter) well polished with alumina paste and sonicated prior to measurement. The counter electrode (CE) was a flame-cleaned platinum mesh at a distance of about 2 cm from the WE, while the tip of the working electrode is 0.5 cm from the WE at the side of the WE–CE current lines. For each electrolyte, the scan was first performed toward cathodic and then anodic potentials. The WE was cleaned between the two scans. The open-circuit potentials were independent from the composition and were approximately −0.10 ± 0.05 V vs RE. The initial potential of LSV was set to zero vs reference for scans on both cathodic and anodic sides. All of the LSV measurements were performed at 25 °C under nitrogen flux using a scan rate of 0.5 mV S^–1^.

## Results and Discussion

**Figure 1:**
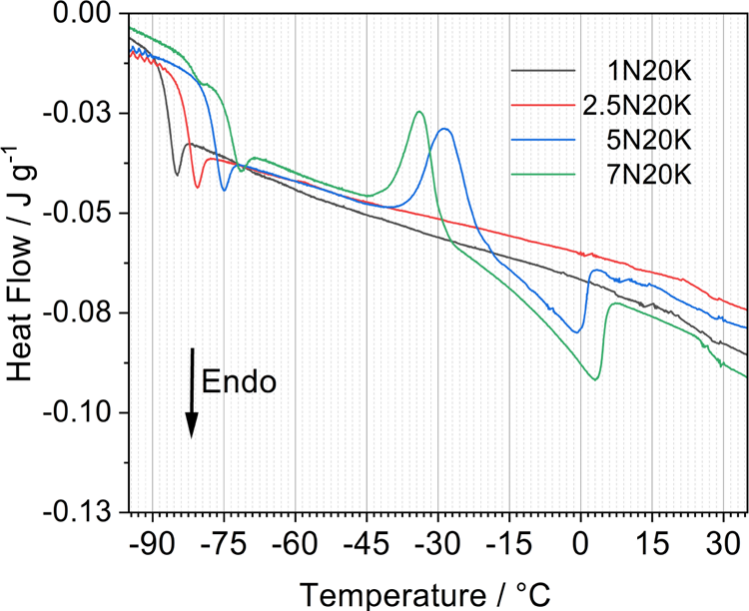
DSC curves of the different electrolytes.

Figure 1 reports the DSC thermograms of the electrolyte solutions. All of the samples show a glass transition, *T*_g_, in the range from −90 to −70 °C depending on the salt concentration (see Table 2). In particular, *T*_g_ increases with the overall salt content (see parameter n* in Table 1). This behavior is correlated to a growing interaction among the solution components, as previously reported for KAc^22^ and LiTFSI^28^ solutions. The *T*_g_ of all of the samples also showed an endothermic overshoot, which can be ascribed to structural relaxation.^29^ Interestingly, the most concentrated sample 7N20K showed an inflection point at approximately −80 °C that points to the existence of two *T*_g_’s assigned to different glassy phases. Above the *T*_g_, the less concentrated samples, 1N20K and 2.5N20K, did not show any other thermal feature, meaning that they remained in the state of supercooled liquid until the expected melting temperature was reached. Indeed, we cannot exclude that cold crystallization could take place for lower heating rates due to the dynamic nature of the DSC measurements. In contrast, at higher NaAc concentrations (5N20K and 7N20K), the DSC traces showed cold crystallization, starting at *T*_c_, followed by melting endotherms, starting at *T*_m_. The onsets of the *T*_c_ were at −46 and −40 °C for the 5N20K and 7N20K samples, while the corresponding *T*_m_ were at −28 and −20 °C, respectively. Table 2 reports the values of melting enthalpy, Δ*H*_m_, and cold crystallization enthalpy, Δ*H*_c_, for the two samples 5N20K and 7N20K. In the case of the sample 5N20K, the two enthalpies are nearly equal, which means that this sample is fully amorphous at low temperatures, i.e., below the glass transition. In the case of the sample 7N20K, in contrast, Δ*H*_m_ is ∼15% greater than Δ*H*_c_, which points to a semicrystalline nature of this sample at low temperatures. This was confirmed by X-ray measurements performed vs. temperature (see Figure S1) that showed the growth of a crystalline pattern below −35 °C together with a broad feature centered at ∼30°, which is attributed to the amorphous phase. The semicrystalline nature of the sample 7N20K was further confirmed by the DSC thermogram obtained during cooling and reported in Figure S2. While the other samples did not show any reversible crystallization, the sample 7N20K displayed a clear exotherm around −35 °C with reversible enthalpy Δ*H*_c,r_ ≈5 J g^–1^. As expected, the sum of this value and the cold crystallization enthalpy reported in Table 2 is in good agreement with the total melting enthalpy of the system. Unfortunately, we were not able to assign the X-ray peaks of Figure S1 to known any crystal phase(s). Further work is needed to solve this problem. Reber et al.^30^ reported liquidus temperatures for several WISEs, such as 35 mol kg^–1^ NaFSI, 30 mol kg^–1^ NaFSI with 5 mol kg^–1^ NaFTFSI, 25 mol kg^–1^ NaFSI with 10 mol kg^–1^ NaFTFSI, and 19.5 mol kg^–1^ LiTFSI with 8.3 mol kg^–1^ LiBETI, at 50, 44, 38, and 28 °C, respectively. They also reported liquidus temperatures of 28 and 22 °C for 35 mol kg^–1^ LiFSI and for 25 mol kg^–1^ LiFSI with 10 mol kg^–1^ LiFTFSI, respectively. In this scenario, the proposed electrolytes in the current work provide a significant advantage by assuring thermodynamically stable liquid phases well below 0 °C.

**Table 2:** Physicochemical Parameters of the Samples^a^

sample name	molarity (mol dm^–3^)	*E*_a_ (η) (eV)	σ (25 °C) (mS cm^–1^)	Λ_m_ (25 °C) (S cm^2^ mol^–1^)	*E*_a_ (σ) (eV)	*T*_0_ (°C)	*T*_g_ (°C)	Δ*H*_m_ (J g^–1^)	Δ*H*_C_ (J g^–1^)
1N20K	9.45	0.23-	49.97	5.29	0.26	–56	–88	N/A	N/A
2.5N20K	9.87	0.24-	41.13	4.16	0.27	–54	–84	N/A	N/A
5N20K	10.38	0.27-	29.75	2.87	0.27	–51	–79	–12.0	12.1
7N20K	10.76	0.29-	21.21	1.97	0.296	–45	–76	–15.3	13.2

a *E*_a_ (η) = activation energy from viscosity measurements, σ = specific conductivity, Λ_m_ = molar conductivity, *E*_a_ (σ) = activation energy from conductivity measurements, *T*_0_ = VTF parameter (see text), Δ*H*_m_ = melting enthalpy, Δ*H*_C_ = crystallization enthalpy.

**Figure 2:**
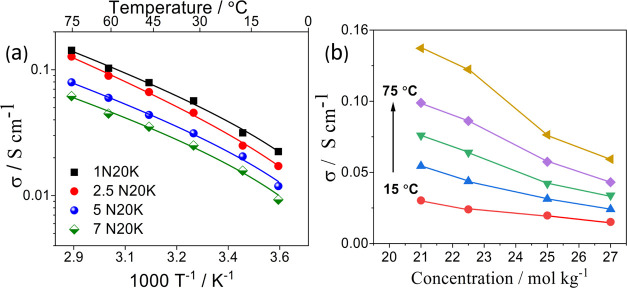
(a) Arrhenius plot of ionic conductivity. Continuous lines correspond to VTF best fits with values of *R*^2^ > 0.99 and (b) conductivity values vs. total salt concentration at different temperatures (15–75 °C, Δ*T* = 15 °C).

Figure 2a shows the dependence of ionic conductivity on temperature for different salt concentrations. Even in the relatively narrow temperature range we explored, all of the compositions follow the Vogel–Tammann–Fulcher (VTF) behavior
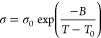
1

Here, *B* is the pseudo-activation energy for ion transport (*E*_a_ = *k*_B_*B*, where *k*_B_ is the Boltzmann constant), *T* is the absolute temperature, and *T*_0_ is an empirical parameter that can be interpreted as the ideal glass-transition temperature. In organic liquids and gel polymer electrolytes, the *T*_0_ value obtained by fitting with eq 1 usually falls 10–50 °C below the kinetic *T*_g_ value measured by DSC, which may be explained in terms of decoupling between the ion motion and lattice structural relaxation.^31^

In contrast, in these WISEs, we found *T*_0_ ≈*T*_g_ + 30 °C (see Table 2) irrespective of the concentration. This points to a strong correlation between the onset of lattice dynamics at the glass transition and the onset of long-range ionic motions likely due to ion–solvent coupling.

The ionic conductivity decreases with increasing the electrolyte concentration (Figure 2b), as expected in highly concentrated electrolytes. All of the sodium-containing samples, indeed, show values lower to that of 20 M CH_3_COOK.^22^ However, the 7N20K sample still exhibits a remarkable conductivity of 21.2 mS cm^–1^ at 25 °C (see Table 2). The behavior of the ionic conductivity with the salt concentration is related to the corresponding shift of *T*_g_ to higher temperatures by increasing the amount of sodium acetate, which determines the increase of viscosity resulting in increased resistance to the shear flow^32,33^ and a higher activation barrier to the movement of ions (see Table 2).^34^ The dependence of *T*_g_ on concentration is attributed to the strong interaction between the ions and the water molecules, suggesting the formation of an internal liquid structure. The considerable levels of conductivity at high concentrations strongly suggest the possibility of the formation of water–salt structures.^32^ Here, the still available water molecules cannot completely shield the solvated cations, so promoting the interaction of anions (CH_3_COO^–^ in our case) with the cations without causing any significant reassociation resulting in the modification of the local hydrogen bonding.^35^ The molar conductivities (Λ_m_), activation energies (*E*_a_), ionic conductivities (σ), and *T*_0_ and *T*_g_ values are reported in Table 2, together with the enthalpy values obtained from Figure 1.

**Figure 3:**
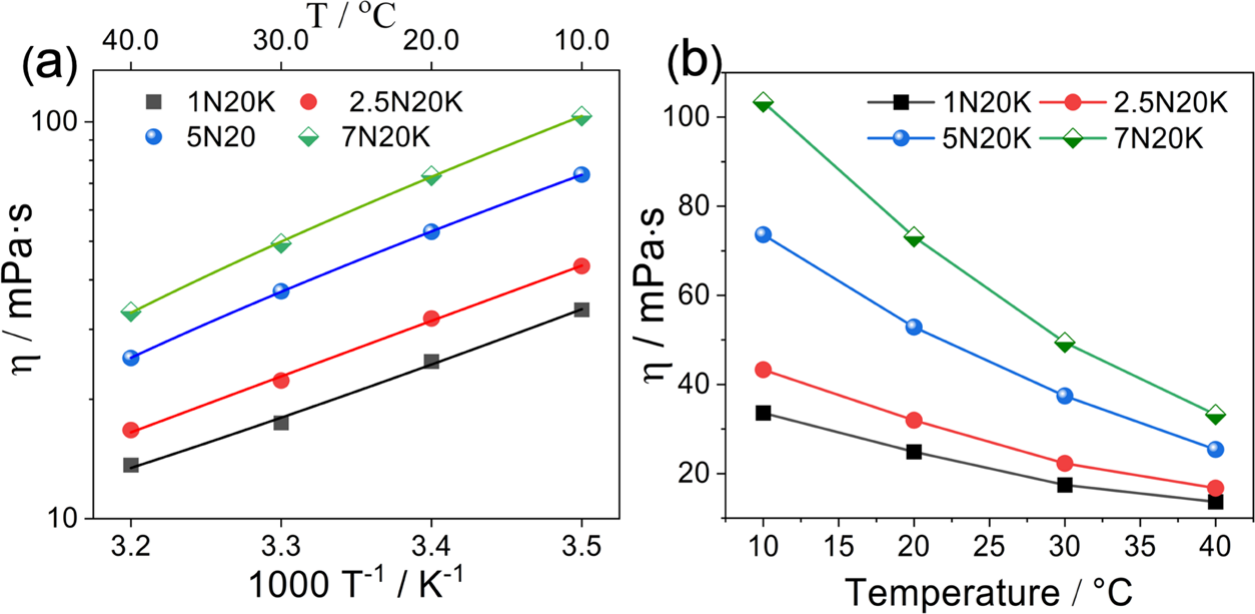
(a) Arrhenius plot of viscosity. The solid lines show linear regression with *R*^2^ > 0.99. (b) Viscosity of all samples vs temperature.

The results on the charge transport are also supported by the viscosity measurements vs the temperature, which follow an Arrhenius behavior (Figure 3a) for all of the electrolyte samples
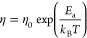
2where *E*_a_ is the activation energy that is related to ion–ion and ion–solvent interactions, while *k*_B_ is the Boltzmann constant. Table 2 reports the *E*_a_ values of the electrolytes calculated from the viscosity data. The activation energy increases with increasing the concentration and the values are in good agreement with those obtained from conductivity measurements. This confirms that the mechanisms at the base of bulk properties such as ionic transport and viscosity are well correlated, in agreement with the thermal results.

All of the electrolyte samples behaved like Newtonian fluids (see Figures S3–S6). As expected, the viscosity increased as a function of concentration. However, it did not vary linearly as it is in the case of diluted solutions due to complex ion interactions at elevated concentrations.^36^ In the case of salts having a similar anion (as in our case), the change in viscosity is predominantly caused by the cations.^37^ Moreover, the behavior of concentrated aqueous acetates rendered them as the so-called “structure-making salts’’ because none of the samples showed any decrease in viscosity with increasing salt concentration. Furthermore, this behavior signifies the reduced activity of water molecules since the cations and acetate anions being strongly hydrated are contributing to some fashion of molecular ordering.^38^

**Figure 4:**
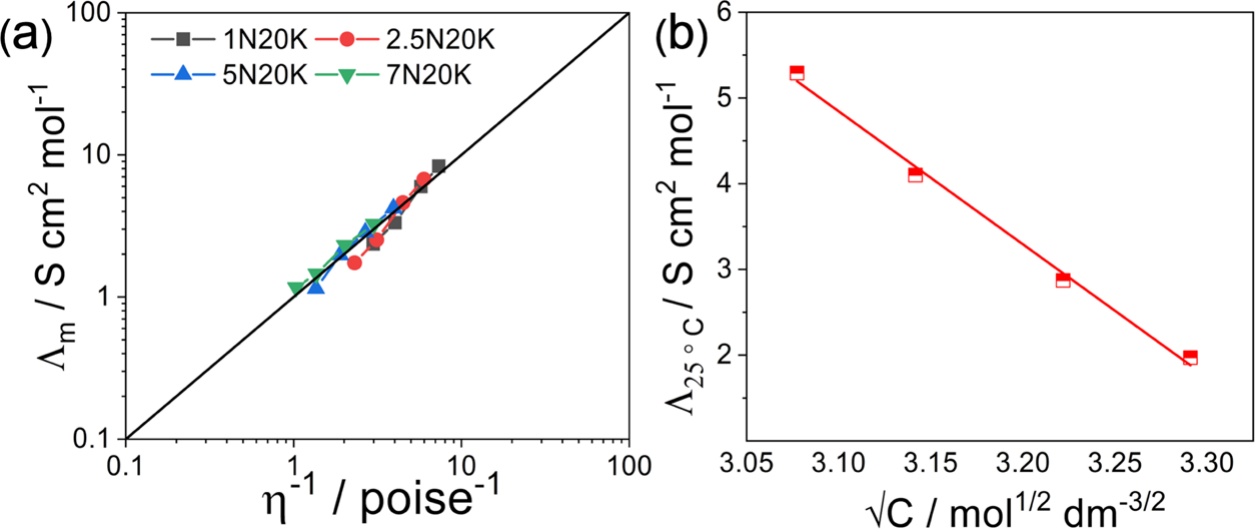
(a) Walden plot for acetate solutions. The straight line indicates the behavior of KCl as a reference. (b) Plot of molar conductivity vs square root of concentration.

Classical Walden plot can be used to probe the ionicity of the acetate electrolytes. Figure 4a shows the molar conductivities vs the fluidity, η^–1^, at different temperatures. The straight line is the ideal line for completely dissociated ionic systems, e.g., KCl-diluted aqueous solutions. The degree of fluidity of the medium is directly related to the association of ions in the electrolyte solutions.^39^ According to Figure 4a, all of the compositions can be classified as quasi-ideal, or good, electrolytes.^40,41^ As expected, the higher the temperature, the better the ideal. In the explored compositional range, the system Na^+^/K^+^CH_3_COO^–^ follows a Kohlrausch-type behavior of strong electrolytes, as reported in Figure 4b, for the values at 25 °C
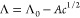
3where Λ is the molar conductivity, Λ_0_ is the intercept at infinite dilution, *A* is a constant independent from the concentration, and *c* represents the molarity (mol L^–1^). At 25 °C, Λ_0_ has a value of 52.90 S cm^2^ mol^–1^, while the constant *A* is 15.50. Thus, the Kohlrausch behavior calls for complete salt dissociation in the whole compositional range.^42^

**Figure 5:**
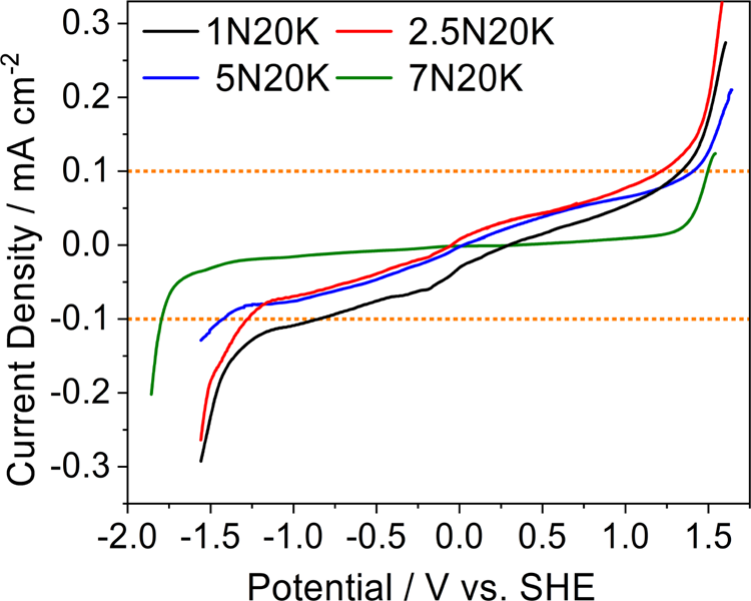
Linear sweep voltammetry of the electrolytes for the determination of their electrochemical stability windows.

Figure 5 shows the linear sweep voltammetry (LSV) profiles vs standard hydrogen electrode (SHE) for the electrolyte samples. For the estimation of decomposition potentials of water, a threshold of 0.1 mA cm^–2^ was taken as a limit on the glassy carbon electrode. The present procedure may lead to overestimated decomposition limits because it involves a large amount of electrolyte and a quasi-ideal electrocatalytic electrode with a small surface area. However, it has the advantages of being highly reproducible and it is useful to compare different electrolytes.

The cathodic and anodic decomposition potentials for all of the samples obtained in these conditions are summarized in Table 3. The noticeable increase in the stability on the cathodic side with increasing salt concentration confirms the suppression of hydrogen evolution. This is a confirmation of the reduced activity of water, in agreement with previous analyses. The ESW expansion on the anodic side is not as significant as it is on the cathodic scan as previously reported for acetate-based electrolytes.^22^ This is due to the combination of two effects: the increase in both pH (see Table S3) and concentration of the acetate ion. In the first case, the concentration of OH^–^ ions increases from 8.7 × 10^–5^ to 1.7 × 10^–4^ M, however, it is reasonable that in the concentrated solution, their activity is lower and inversely proportional to the salt concentration. Then, acetate ions should be involved in the anodic decomposition process and it is reasonable that the onset of the decomposition potential decreases as the concentration increases.

**Table 3:** Cathodic and Anodic Stability Limits, ESW

sample name	cathodic limit (V)	anodic limit (V)	ESW (V)
1N20K	–0.84	1.33	2.17
2.5N20K	–1.27	1.21	2.48
5N20K	–1.42	1.40	2.82
7N20K	–1.79	1.49	3.28

**Figure 6:**
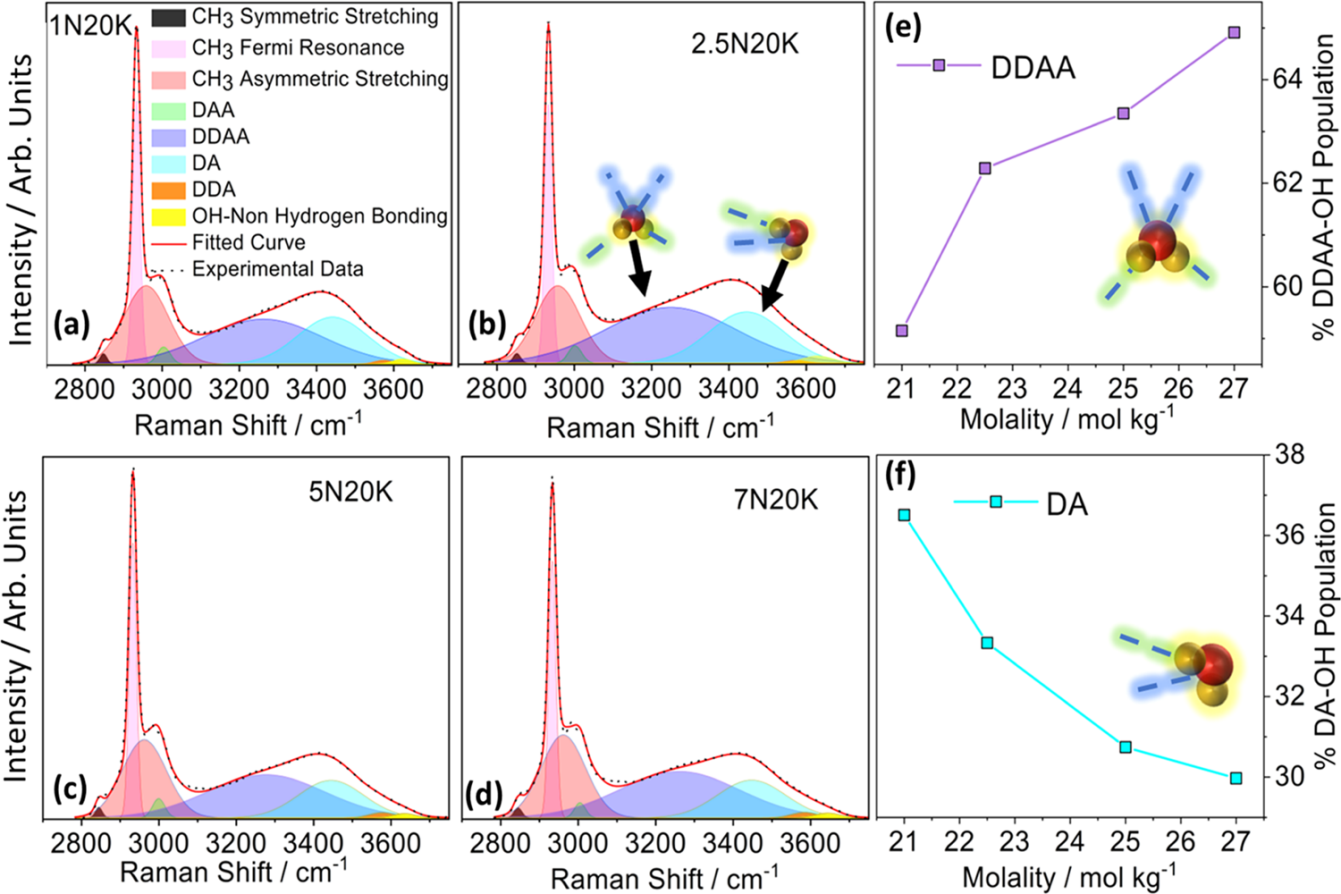
Raman spectra in the OH region for different NaAc concentrations (a–d) and analyses of DDAA and DA contributions (e, f).

To better understand the interactions between the components of the solution and to correlate the structure with the functional properties, Raman analysis vs. both sodium acetate concentration and temperature was performed. Raman study vs electrolyte concentration was carried out at room temperature to understand the evolution of hydrogen bonding in these highly concentrated electrolytes. A deeper insight into the local hydrogen-bonding network is key to understanding the peculiar properties of water in such solutions. Thus, we focused our analysis on the high wavenumber region of the Raman spectrum (i.e., 2800–3800 cm^–1^) as reported in Figure 6a–d. In fact, this is the region where the main OH-related bands fall, which showed strong dependence on the composition. The region below 2800 cm^–1^, which includes all of the main vibrational modes of the acetate group, did not vary significantly with the cation composition and will be deeply discussed in the section devoted to the temperature evolution of the spectra. However, for the sake of completeness, the entire Raman spectra are reported in Figure S7 for all of the investigated compositions. In the high wavenumber region, based on the behavior of a specific water molecule as either a proton donor (D) or acceptor (A) in a hydrogen bond, the characteristic OH stretching band can be deconvoluted into five sub-bands, namely, DAA, DDAA, DDA, DA, and nonhydrogen bonding motifs located at ∼3000, ∼3300, ∼3450, ∼3600, and ∼3650 cm^–1^, respectively^26,43,44^ (see Figure S8 for the sketch of the different interactions). In the same spectral region, some acetate vibrations can be assigned, namely, the CH_3_ symmetric stretching (CH_3_:SS) at ∼2845 cm^–1^, the CH_3_ Fermi resonance (CH_3_:FR) at ∼2935 cm^–1^, and the CH_3_ asymmetric stretching (CH_3_:AS) at ∼2981.^45−47^ The spectra were fitted as a sum of CH_3_- and OH-related peaks for a total of eight Gaussian bands as described above (the fitting results are reported in Tables S1 and S2). It is important to underline that all five OH stretching vibrations are present even at very high concentrations, including contributions from both water–water and water–solute interactions. Minor changes in the position and in the full width at half-maximum (FWHM) of the bands were registered. However, they are within experimental and fitting errors and, apparently, they do not follow a clear trend as a function of salt concentration. In contrast, band intensities were strongly influenced by acetate concentration. Considering the relative intensities of the different OH bands, a large part of the signal came from the DDAA and the DA bands which, together, were responsible for 95% of the total peak area. Focusing attention on these two peaks, as shown in Figure 6e,f, it emerged clearly that a higher concentration of acetate led to a lowering of the DA population in favor of the DDAA counterpart. The DA band corresponds to water with weak hydrogen bonds, whereas the DDAA corresponds to hydrogen bonds with high binding energies and shorter bond lengths.^48^ In fact, the hydrogen bond strength is inversely related to the frequency; therefore, the bands at lower or higher frequencies are attributed to stronger or weaker hydrogen bonding, respectively.^49^ It can be assumed that at higher concentrations, the interaction of water with the acetate anion is more favored than the water–water or water–cation interactions. Hence, water molecules are effectively seized by the acetate anions present in such a high population, where the carboxylic group is accepting the hydrogen bonds in a bidentate-like structure. These findings may explain the electrochemical behavior of the different solutions. In fact, there is a direct correlation between the DDAA population and the ESW, in particular with the cathodic decomposition potential. An increase in the overpotential for water degradation is due to the reduced activity of water because of strong hydrogen bonding with the carboxylate group of the acetate anion.^26,49^

**Figure 7:**
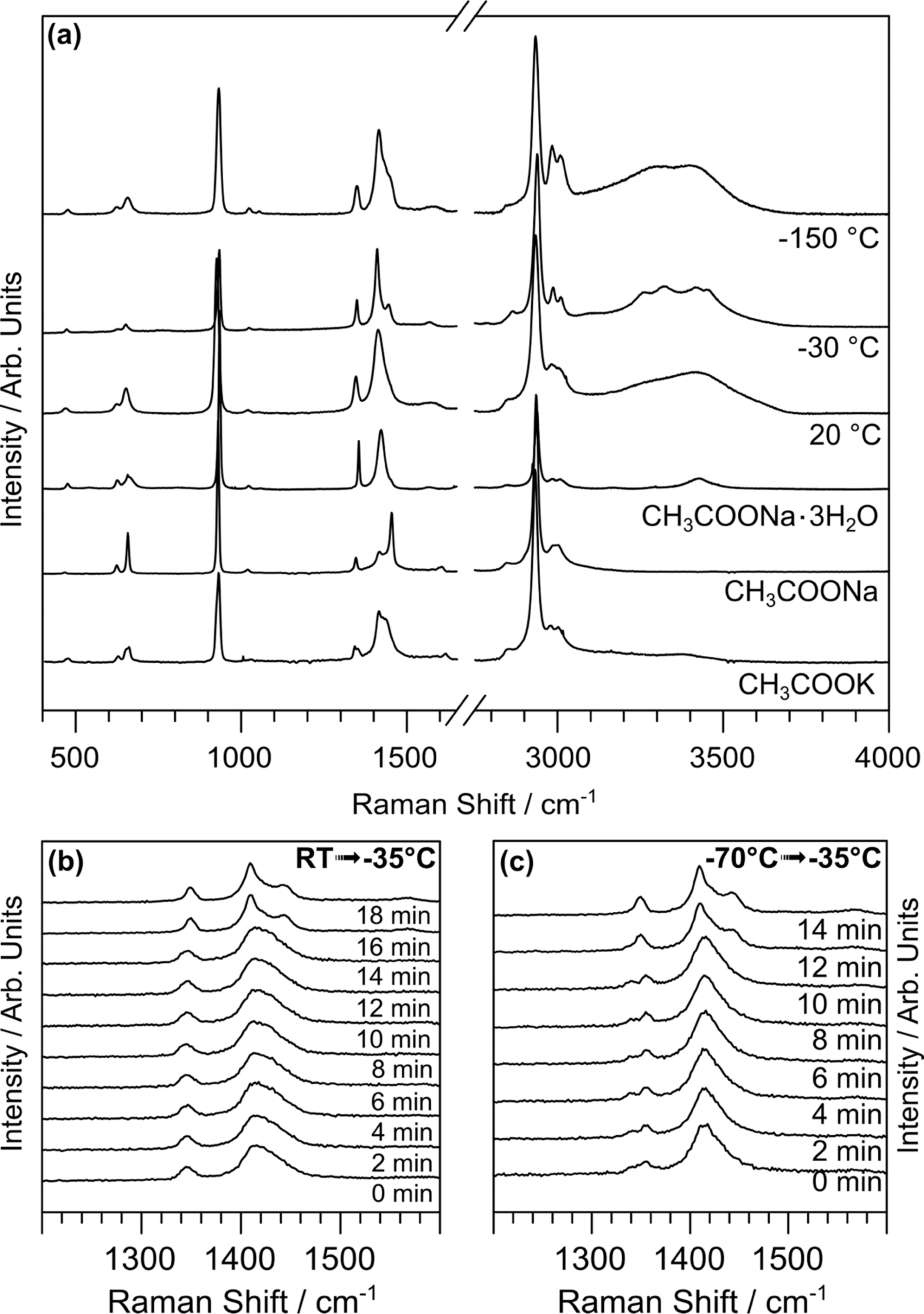
Raman spectra as a function of temperature (a) and time (b, c) for the 7N20K electrolyte. The experiment reported in panel (b) was performed by starting at *T* = −70 °C, then heating at −35 °C, and acquiring a spectrum every about 2 min when the target temperature was reached. The experiment reported in panel (c) was performed by starting at room temperature, cooling at −35 °C, and acquiring a spectrum about every 2 min when the target temperature was reached.

To further characterize the solution structure and to better explain the thermal behavior of the system (DSC curves in Figure 1), Raman analysis was performed at different temperatures, even if by limiting the analysis to the extreme of the series (1N20K and 7N20K). Figure 7 shows three representative spectra collected at −150, −30, and 20 °C for the sample 7N20K (see Figure S9 for the whole spectra). Similar results were obtained for the sample 1N20K (see Figure S10). All of the spectra presented the expected features of aqueous solution of acetate salts even though clear changes could be appreciated as a function of temperature.

The main vibrational modes of the acetate group are the COO rocking (below 750 cm^–1^), the C–C stretching (926 cm^–1^), the CH_3_ deformation (1345 cm^–1^), and C–O stretching (1413 cm^–1^), while water- and CH_3_-related stretching bands occur above 3000 cm^–1^ as discussed previously. The thermal evolution of the Raman spectra could be grouped into three major families, respectively, amorphous glass-like state at *T* < −50 °C, crystalline state at −50 °C < *T* < 10 °C, and liquid state at *T* > 10 °C, in good accordance with the DSC signals. The spectral features most affected by the change pertained both to the acetate and water groups. In particular, passing from the liquid to crystal phase, we observed the sharpening of the main C–C stretching accompanied by a shift from 926 to 931 cm^–1^. At the same time, the C–O stretching mode also sharpened with the emergence of a second peak at 1443 cm^–1^. The small peak due to the methyl deformation also became sharper and registered a small shift passing from 1345 to 1348 cm^–1^. The overall peak sharpening points toward the formation of a crystal structure. Thus, we compared our data with the Raman spectra of prototypical crystals of acetate salts to figure out the arrangement of the metastable crystal phase (Figure 7a). The salts taken into consideration are CH_3_COONa and CH_3_COONa_3_·3H_2_O, which crystallize in orthorhombic and monoclinic systems, respectively.^50,51^ The positions and widths of the Raman peaks in the liquid phase were very similar to those of CH_3_COONa_3_·3H_2_O, slightly modified by the presence of the potassium . This is consistent with data collected on droplets of sodium acetate solutions at different water-to-solute ratios,^52^ where the observed Raman modes are very similar to the trihydrate form, at least for lower concentration. In the trihydrate form, the Na^+^ ions are octahedrally coordinated by oxygen atoms of water and acetate molecules, forming chains of edge-sharing octahedra aligned along the *z*-axis.^51^ Thus, the structure of the liquid phase probably consists in a random network of octahedrally coordinated cations. At the crystallization temperature, the new peak appearing at 1443 cm^–1^, as well as the sharpening and shifting of the other peaks, suggested the formation of compact ordered structures featuring spectral characteristics similar to anhydrous CH_3_COONa crystals. These salts, in the crystalline form, consist of a layered structure alternating cation-carboxylate and methyl group layers.^50^ Simultaneously, the OH region showed a pronounced separation of the DA and DDAA bands accompanied by a sharpening of the two bands, suggesting an ordering also of the hydrogen bonds. Importantly, after the re-organization of water molecules, the appearing Raman spectrum is distinct from any common form of ice and points toward a structure with short-range order as in liquid water.^53^ Finally, above the melting temperature, the spectra turned out to be similar to the aqueous acetate solution collected at low temperatures, i.e., below the glass transition. As in the liquid state, the glassy state preserves a random distribution of octahedrally coordinated cations but with a disordered distribution of bond angles, as suggested by the widening of the main peaks. We remark that, in contrast to DSC experiments, also the sample 1N20K showed the same behavior as a function of temperature with the occurrence of the first steps of cold crystallization at *T*_C_ ≈ −20 °C (see Figure S8). Indeed, this can be due to the different experimental protocols of DSC and Raman measurements, reflecting the kinetic nature of cold crystallization.

Finally, the crystallization kinetics of the sample 7N20K was isothermally monitored in the 1200–1600 cm^–1^ region both by passing from room temperature to −35 °C and from *T* = −70 °C up to −35 °C, where the crystallization was expected to take place in a matter of minutes (see DSC results). As summarized in Figure 7b,c, the crystallization could be reached both during cooling and heating processes with similar kinetics of the order of 15 min.

## Conclusions

In this work, the physicochemical properties of a series of binary aqueous acetate salt solutions were thoroughly investigated via thermal, rheological, electrical, electrochemical, and spectroscopy measurements. Four different solutions were prepared by dissolving 20 mol kg^–1^ CH_3_COOK, with different amounts of CH_3_COONa, ranging from 1 to 7 mol kg^–1^. DSC analysis carried out in a temperature range of −120 to 90 °C revealed that the glass-transition temperature *T*_g_ increases with increasing the salt concentration. Moreover, the DSC also confirmed the low liquidus temperature for the most concentrated 7N20K sample. Viscosity measurements carried out vs. temperature classified the acetate salts to belong to the so-called “‘structure-making salts”’. Ionic conductivity decreased by increasing salt concentration. However, the remarkable value of 21.2 mS cm^–1^ at 25 °C was obtained for the 7N20K most concentrated sample. Electrochemical measurements showed that the electrochemical stability window increased by increasing the CH_3_COONa concentration. Such an increase is due to the shift of cathodic decomposition to more negative potentials, while the anodic decomposition limit is less affected by the salt concentration. The peculiar interaction among phase components and the emerging structural features were deeply investigated by Raman spectroscopy, which shed light on the evolution of hydrogen-bonding motifs in these concentrated solutions. A careful Raman analysis conducted vs. temperature helped in tracing out a possible structure of the crystal formation during the crystallization process. Due to the good ionic conductivity, large ESW, and low liquidus point, these concentrated systems are promising as WISEs for batteries and supercapacitors.

## Supporting Information Available

Shear stress vs. shear rate curves for aqueous electrolytes, Raman and thermal analysis (PDF)

## Abbreviations

LiBs: lithium-ion batteries
ESW: electrochemical stability window
NaAc: sodium acetate
KAc: potassium acetate
