# Supplementary material for: Structure–Property Correlations in Aqueous Binary Na+/K+–CH3COO– Highly Concentrated Electrolytes
Source: J Phys Chem C Nanomater Interfaces. 2023 May 13;127(20):9823–32. doi: 10.1021/acs.jpcc.3c01017 (PMC10226112; doi:10.1021/acs.jpcc.3c01017)
Supplement: Supplementary file 1 — jp3c01017_si_001.pdf [file jp3c01017_si_001.pdf]

Supporting Information

Structure-Property Correlations in Aqueous Binary Na^{+}/K^{+}-CH_3COO^{-} Highly Concentrated Electrolytes

*Shahid Khalid[a], Nicolò Pianta[a], Simone Bonizzoni[a], Chiara Ferrara[a,b], Roberto Lorenzi[a], Alberto Paleari[a], Patrik Johansson[b], Piercarlo Mustarelli[a,c], Riccardo Ruffo[a,c]**

[a]Department of Materials Science, University of Milano-Bicocca, via Cozzi 55, 20125, Milano, Italy

[b]Department of Physics, Chalmers University of Technology, SE-41296 Göteborg, SWEDEN

[c]National Reference Center for Electrochemical Energy Storage (GISEL) - Consorzio Interuniversitario Nazionale per la Scienza e Tecnologia dei Materiali (INSTM), 50121 Firenze, Italy

KEYWORDS: water-in-salt, aqueous electrolytes, sodium ion batteries, electrochemical stability window, potassium acetate, sodium acetate

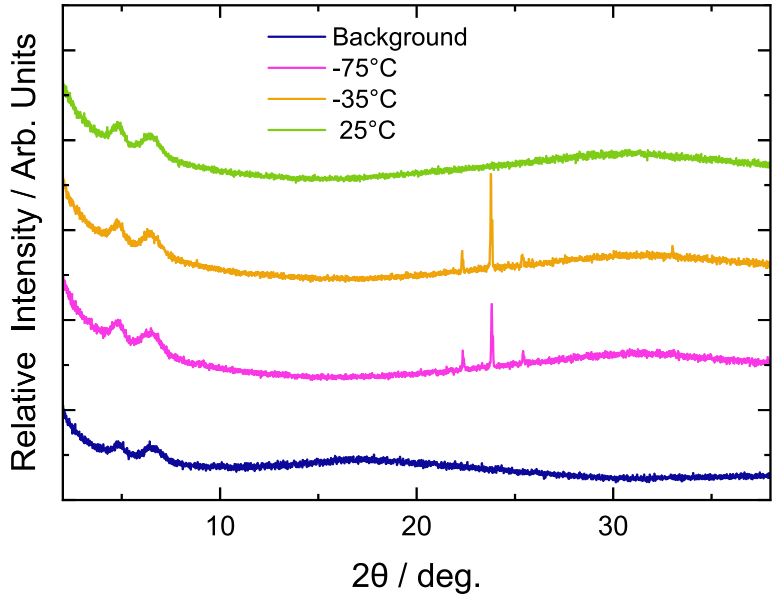

Figure S1. XRD patterns of 7N20K obtained at different temperatures.

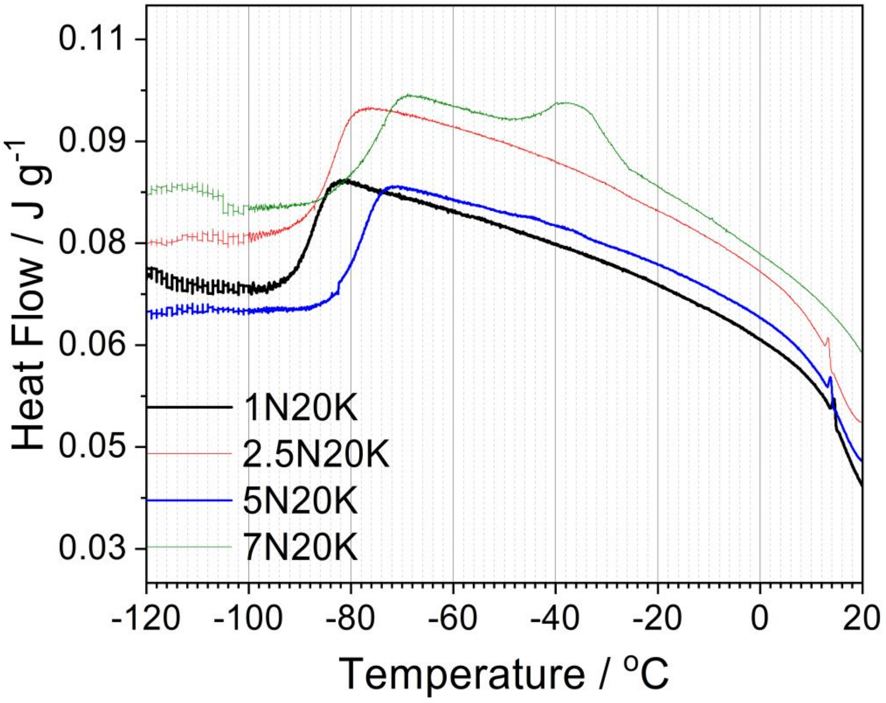

Figure S2. DSC profiles during the cooling for the different electrolytes

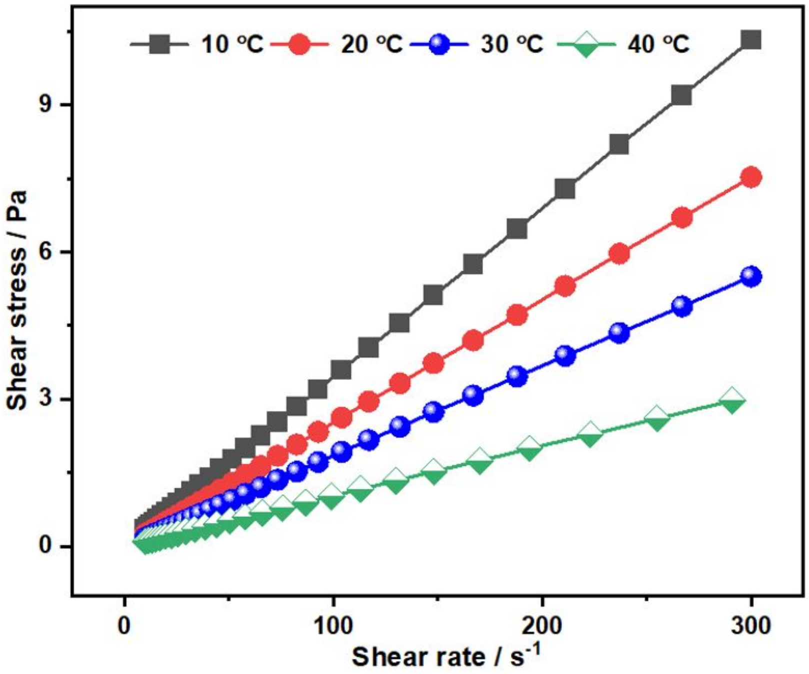

Figure S3: Curves of shear stress vs shear rate for 1N20K

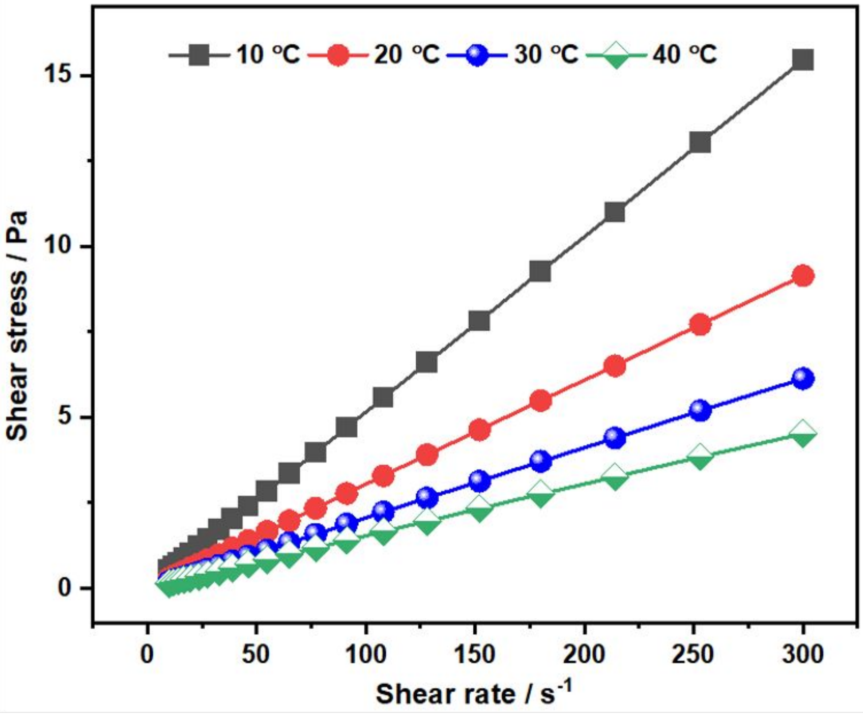

Figure S4:Curves of shear stress vs shear rate for 2.5N20K

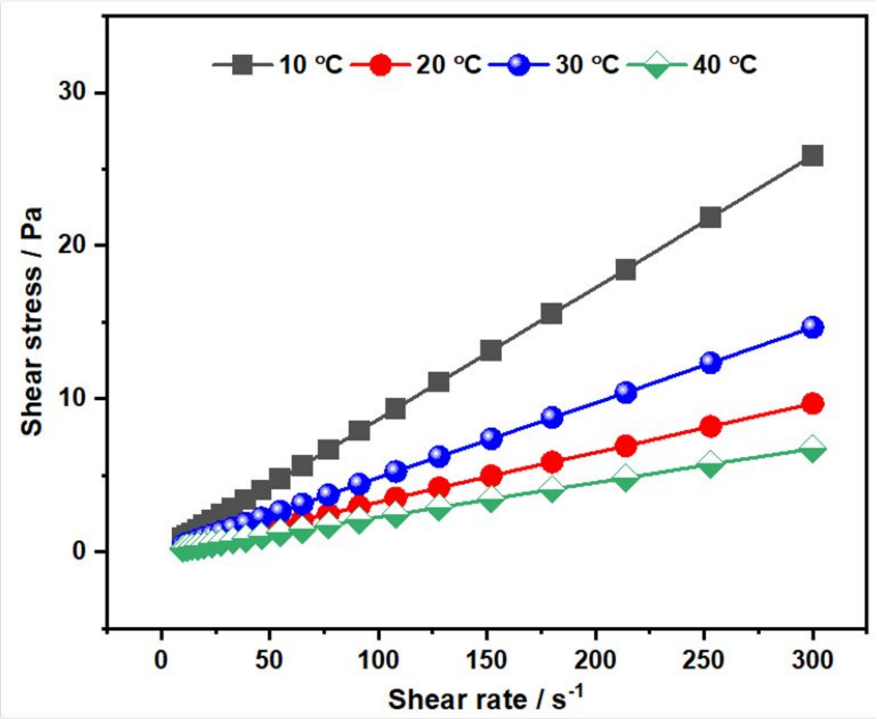

Figure S5: Curves of shear stress vs shear rate for 5N20K

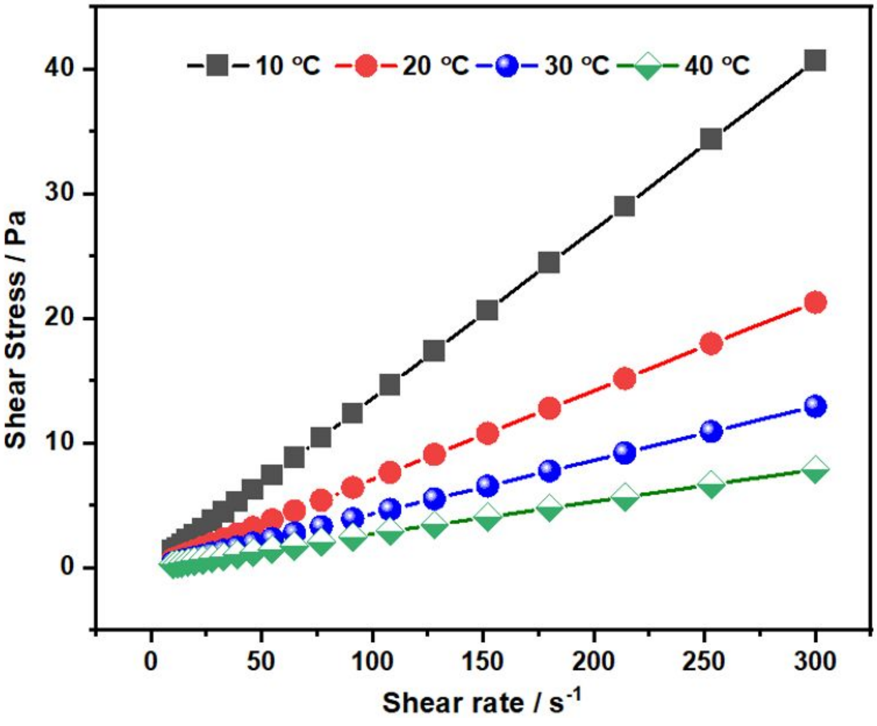

Figure S6: Curves of shear stress vs shear rate for 7N20K

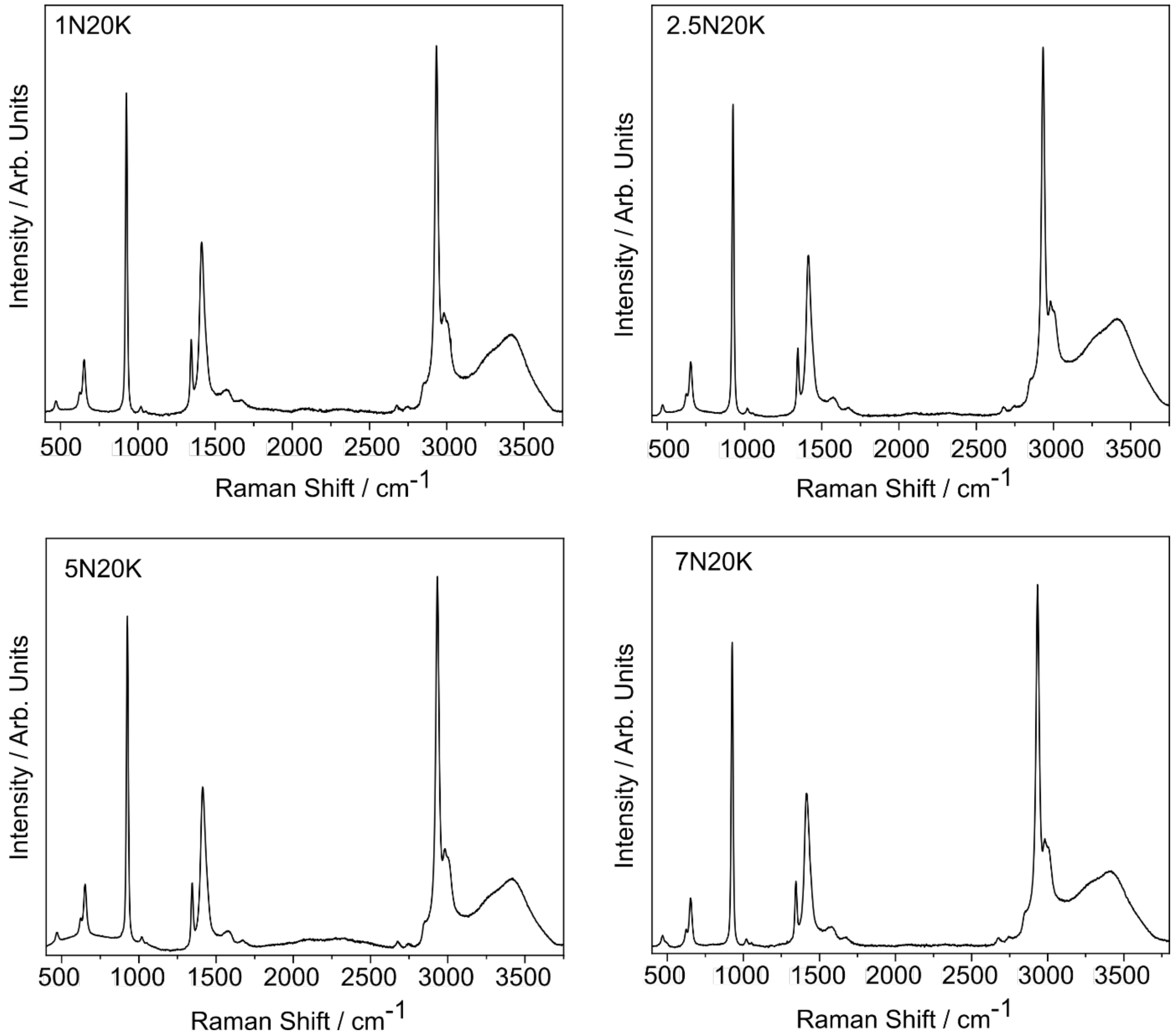

Figure S7. Raman spectra at room temperature for the different electrolytes

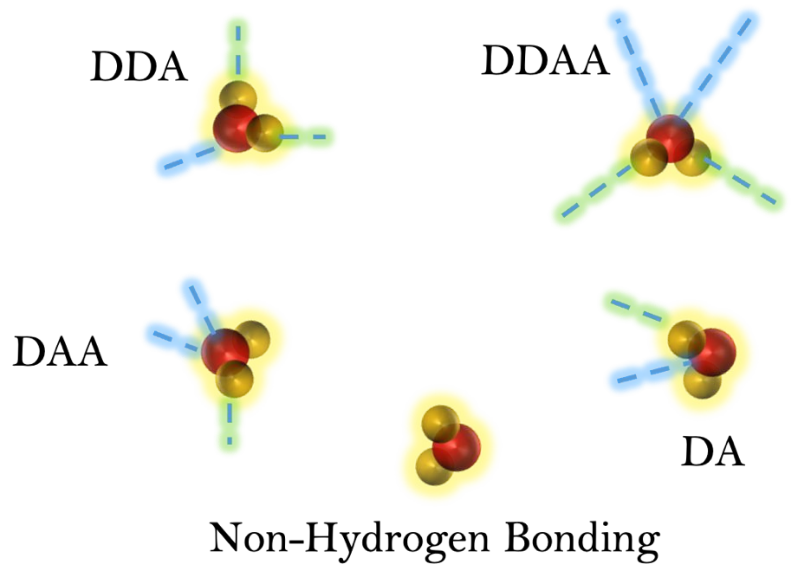

Figure S8. Graphical representation of sub-bands DAA, DDAA, DDA, DA, and non-hydrogen bonding of OH stretching region from ~3000 cm^{-1}.

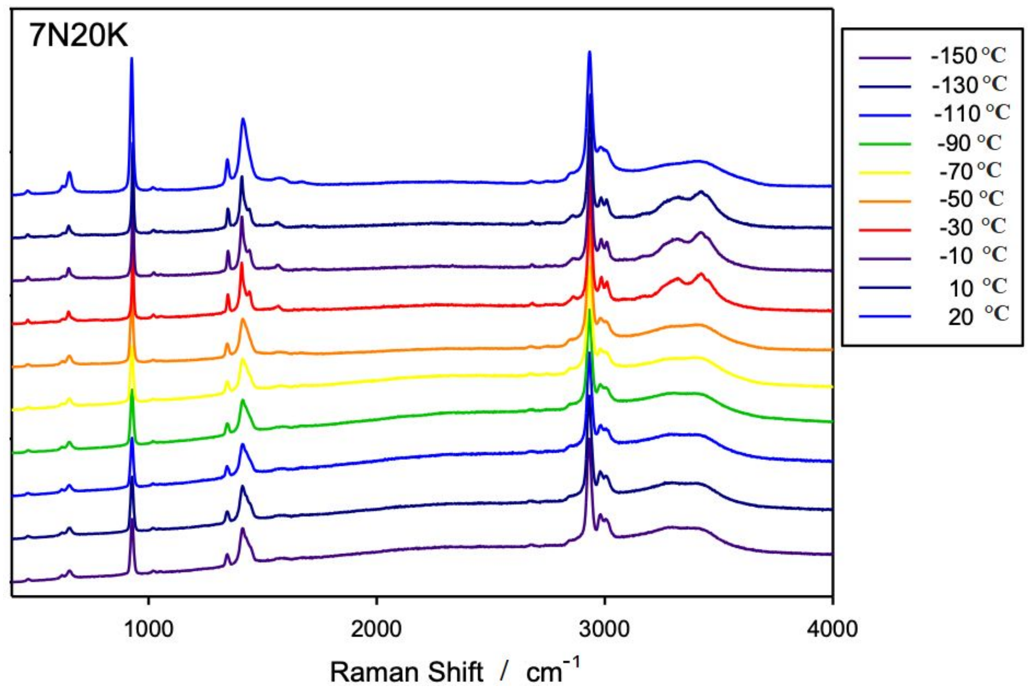

Figure S9: Full spectra as a function of T for 7N20K

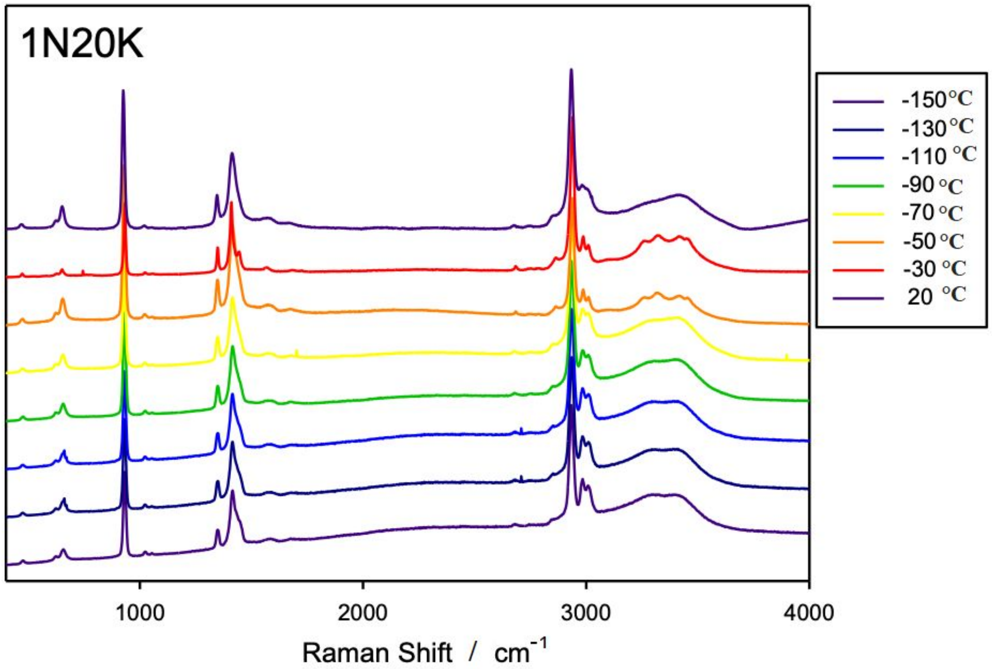

Figure S10: Full spectra as a function of T for 1N20K

Table S1: Fitting results of Raman spectra decomposition in Gaussian functions in the OH region:

	1N20K			2.5N20K			5N20K			7N20K		
	**AREA**	**FWHM (cm-1)**	**Centre (cm-1)**	**Area**	**FWHM (cm-1)**	**Centre (cm-1)**	**Area**	**FWHM (cm-1)**	**Centre (cm-1)**	**Area**	**FWHM (cm-1)**	**Centre (cm-1)**
**CH_3 SYMMETRIC STRETCHING**	17169	22	2848	18288	24	2852	18314	22	2845	18424	25	2845
**CH_3 FERMI RESONANCE**	443141	22	2934	471207	22	2933	490563	22	2933	416212	22	2933
**CH_3 ASYMMETRIC STRETCHING**	802674	136	2959	857962	137	2958	857722	135	2962	830131	136	2962
**DAA-OH**	51219	40	3004	57724	41	3002	59416	38	3000	37906	34	3005
**DDAA-OH**	1215950	360	3262	1790813	397	3250	1248162	357	3278	1251965	366	3262
**DA-OH**	750541	213	3442	958287	231	3446	605770	199	3445	578033	207	3448
**DDA-OH**	16249	57	3575	14021	66	3575	33524	79	3579	33870	83	3586
**OH-NON HYDROGEN BONDING**	21759	58	3624	54159	95	3630	23424	67	3635	26909	77	3644

Table S2: Relative OH population (in percentage) calculated from the area under the corresponding Raman peak for all the investigated samples.

	POPULATION %			
	**1N20K**	**2.5N20K**	**5N20K**	**7N20K**
**DAA-OH**	2.5	2.0	3.0	2.0
**DDAA-OH**	59.1	62.3	63.3	64.9
**DA-OH**	36.5	33.3	30.7	30.0
**DDA-OH**	0.8	0.5	1.7	1.8
**OH-NON HYDROGEN BONDING**	1.1	1.9	1.2	1.4

Table S3: pH values of the electrolyte samples and decomposition potentials of water at room temperature calculated using Nernst Equations

SAMPLE	PH	CATHODIC STABILITY LIMIT OF H_2O	ANODIC STABILITY LIMIT OF H_2O
**1N20K**	9.94	-0.586	0.644
**2.5N20K**	10.01	-0.596	0.634
**5N20K**	10.04	-0.603	0.627
**7N20K**	10.23	-0.614	0.616
